# Corrosion Monitoring of Reinforced Steel Embedded in Cement Mortar under Wet-And-Dry Cycles by Electrochemical Impedance Spectroscopy

**DOI:** 10.3390/s20010199

**Published:** 2019-12-30

**Authors:** Je-Kyoung Kim, Seong-Hoon Kee, Cybelle M. Futalan, Jurng-Jae Yee

**Affiliations:** 1 University Core Research Center for Disaster-free and Safe Ocean City Construction, Dong-A University, Busan 49315, Korea; kjktit@dau.ac.kr (J.-K.K.); cmfutalan@gmail.com (C.M.F.); 2 Department of Architectural Engineering, Dong-A University, Busan 49315, Korea

**Keywords:** alternative current impedance, cement mortar, corrosion, current uniformity, wet–dry cycles

## Abstract

The primary objective of the present work is to measure the corrosion rate of reinforcing steel embedded in concrete structures in a simulated marine environment of high chloride concentration. The selection of a single frequency that corresponds to the solution resistance and single frequency that corresponds to the charge transfer resistance were performed and measurements were carried out in a relatively faster time. A total of seven cement mortar specimens were prepared. The effect of varying cover thickness (5–50 mm) and rebar distance (10–80 mm) on the electrical resistance of the concrete and corrosion rate of the steel was examined. To simulate the corrosion of reinforced concrete in a marine environment, cement mortars were exposed to 25 wet–dry cycles that involve an immersion for 8 h in 3 wt.% NaCl solution and drying time of 16 h under room temperature. Alternative current (AC) impedance measurements were carried out within a frequency range from 100 kHz to 1 mHz. Results show that the formation of rust layers on rebars has caused a significant decrease in the maximum phase shift to θ = −30°. An accelerated corrosion rate of the rebars was observed during drying stage.

## 1. Introduction

The deterioration of civil infrastructures worldwide is mainly induced by steel bar corrosion in concrete [1]. Reinforced concrete structures that have been degraded by environment-induced corrosion are considered hazardous and unstable that could lead to safety problems and unwanted incidents [2]. The occurrence of corrosion in reinforced structures is extremely high in the marine atmospheric environment. Several factors including high-temperature, relatively high humidity and high NaCl concentration contribute to the corrosion of reinforced structures in the marine environment [3,4,5]. The tide ebbing and flooding expose the reinforced structure via soaking in seawater, seawater film covering the structure during wet state and dry state. Moreover, the repetitive alternating wet–dry exposure at high-frequency in tidal zones will lead to an accelerated corrosion of the reinforced structures [6]. Therefore, it is essential to monitor and understand the corrosion state of the reinforced concrete for structure maintenance.

Electrochemical methods are commonly applied to assess and monitor the corrosion of reinforced concrete and cementitious materials [7]. Several corrosion monitoring methods include polarization curve, half-cell potential, multi-electrode array, electrical resistivity, electrochemical noise, and polarization resistance. These technologies are rapid, inexpensive, highly reliable, and cause less structural damage during application [8]. The measurement of the corrosion potential determines the severity of the corrosion condition of the reinforced concrete. Corrosion potential is affected by several structural (stress, roughness, groove, and shape) and environmental (ventilation, temperature, moisture content, and salinity) factors. Most of the methods can only be applied on a laboratory scale that would imply an inaccurate determination of the on-site information on rebar corrosion such as imprecise assessment of corrosion rates [9]. In theoretical and experimental terms, a significant correlation has been established between the polarization resistance and corrosion current density. The polarization resistance has been determined to be useful in assessing corrosion rates and can measured in an alternative current (AC) or DC mode. The monitoring of AC impedance involves the analysis of polarization resistance via extrapolation of the impedance value to frequency f→0 [10].

The alternative current (AC) method, otherwise known as electrochemical impedance spectroscopy (EIS), assesses numerous electrochemical systems based on the characteristics of electrolytic activities [8]. The properties of resistance (or impedance) and frequency assist in the determination of the corrosion state of reinforced concrete. The AC method is applicable to sites that are difficult to analyze by direct current signal. It is known to be useful in understanding both corrosion rate and various electrochemical phenomena. The charge transfer resistance is also determined from the impedance at a low frequency range. One of the advantages of the AC method is the accuracy in the identification for each reaction phenomena occurring at the metal/solution interface based on the frequency range [11]. The AC method has the ability to evaluate the corrosion morphology and state of the rebar. Moreover, it allows the continuous monitoring of the reinforced concrete in its passive or active state [8]. The components of the solution resistance can be separated at high frequency region, which would make the correction on the dynamic voltage (IR) drop possible. Therefore, analysis of the corrosion mechanism is performed and identification of the cause of corrosion (concentration, diffusion, and activation) based on frequency and speed can be carried out. It is essential to accurately measure the corrosion rate of rebars in concrete that is continuously exposed to the wet and dry process, which increases the electrolyte resistance. The occurrence of corrosion on metal surfaces is categorized as a non-stationary system. With passing time, oxides on metal surfaces form that cause changes in corrosion potential and corrosion rate. A conventional EIS measures the corrosion rate on a continuously changing surface by setting the corrosion potential or fixed voltage at the start of the experiment. Dynamic EIS utilizes a separate reference electrode to measure the corrosion rate of oxides on the metal surface using different potential, which would focus on the change in corrosion behavior over time [12]. The group of Tsuru and Nishikata has used two-electrode cell type to monitor corrosion rates using the same component and size material for both working electrode and reference/counter electrode. The rates of non-stationary corrosion system are measured under various corrosion environments including atmospheric condition [10,13,14,15,16,17,18].

The purpose of this study is to evaluate the capacity of EIS for fast and accurate measurement of the corrosion in reinforced concrete structures that have been repeatedly exposed to seawater immersion and drying. The factors affecting the non-uniformity of the current distribution and IR drop on the surface of the reinforced bar (rebar) were evaluated. To simulate the marine environment, mortar specimens are embedded with rebars and exposed to repeated cycles of immersion in 3 wt.% NaCl solution and drying at room temperature. The corrosion rate of the rebars was monitored using two selected frequencies for high-speed measurement.

## 2. Materials and Methods

### 2.1. EIS Model of Reinforced Steel in Concrete

**Figure 1:**
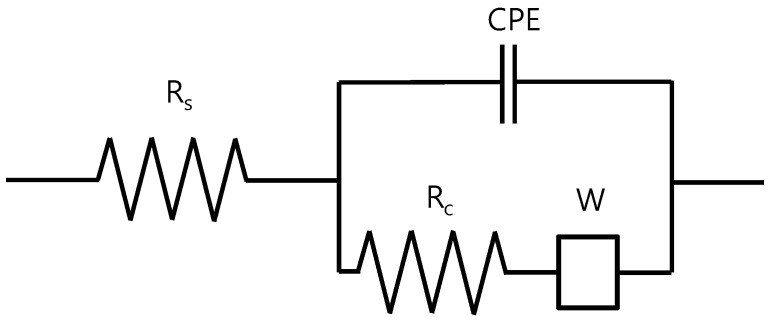
Equivalent electrochemical impedance spectroscopy (EIS) model of a reinforced steel in concrete [10].

The frequency domain is applied in an EIS method such that the interface of the rebar embedded in concrete can be represented as an equivalent electric circuit comprised of resistance, inductance, and capacitance [8]. In this study, the EIS equivalent circuit model consists of solution resistance (R_s_), charge transfer resistance (R_c_), constant phase element (CPE), and Warburg impedance (W; Figure 1). R_s_ represents the electrical resistance generated along the electrolyte solution found within the cement void. R_c_ refers to the resistance of the charge transfer involved in the corrosion mechanism. The electric double layer capacity (C_dl_) refers to the double layer capacity at the interface between cement mortar and rebar while W refers to the diffusion caused by corrosion.

The total impedance (Z) are represented by Equation (1):

(1) $$Z=R_{S}+\frac{R_{c}}{1+j\omega C_{\mathrm{dl}}R_{c}},$$

where j is a complex number that is determined by the frequency response analyzer and ω is the frequency. At high frequencies (ω→∞), the capacitor impedance is 1/ω C_dl_→0 that would result in the measurement of R_s_. Meanwhile, R_s_ + R_c_ is measured at low frequency (ω→0). In other words, R_p_, a polarization resistance, is the difference between the high and low frequency impedance as shown in Equation (2).

(2) $$R_{P}=Z_{\text{low\_frequency}}-Z_{\text{high\_frequency}}.$$

The Stern–Geary equation (Equation (3)) was used to obtain the corrosion current density (i_corr_) from the polarization resistance [19]

(3) $$i_{\mathrm{corr}}=\frac{k}{R_{P}},$$

where k is a proportional constant and computed using Equation (4):

(4) $$k=\frac{b_{a}b_{c}}{2.303(b_{a}+b_{c})},$$

where b_a_ and b_c_ are the Tafel gradients of the positive and negative polarization curves, respectively. In the current study, the value of k = 0.025 V/dec has been applied [20]. 

### 2.2. Preparation of Specimens 

**Figure 2:**
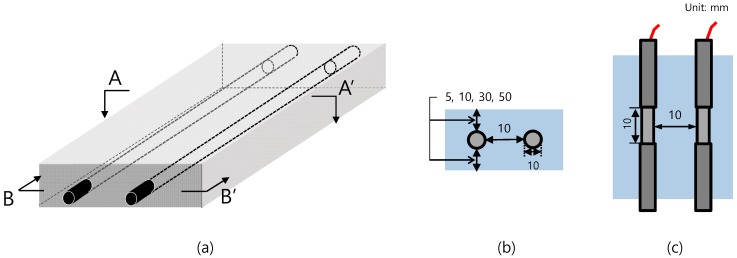
The (**a**) isometric view, (**b**) sectional view A–A’, and (**c**) sectional view B–B’ of the specimen configuration of reinforced steel in a concrete specimen.

The configuration of the concrete specimens is shown in Figure 2. Two rebars of 10 mm in diameter and 100 mm in length were embedded at the center of the cement mortar sample. Each rebar was insulated with a 45-mm heat-shrink tube and B-coating agent on both ends and 10 mm of length was exposed to allow direct attachment to the mortar. The area exposed to concrete for each rebar was fixed at 6 cm^2^. The cement mortar specimens were prepared using Portland cement and ISO standard yarn with a water-cement ratio of 0.6 and cement-sand ratio of 0.5. As shown in Table 1, a total of seven specimens were fabricated under varying cover thickness (5–50 mm) and rebar spacing (10–80 mm). The rebars were fixed by drilling the mold before placement of the concrete.

### 2.3. Wet–Dry Cycle 

A wet–dry cycle refers to the process of immersing the specimen in 3 wt.% NaCl solution for 8 h and dried under room temperature for 16 h. A total of 25 wet–dry cycles was performed in this study. The NaCl solution was prepared using analytical grade NaCl (99.0% purity, Daejung, South Korea) and distilled water. 

AC impedance measurements were performed to determine the electrical resistance of concrete and steel corrosion rate using the multichannel electrochemical workstation (Won-A tec, ZIVE MP2) with an amplitude of ±10 mV. The impedances were measured using the equipment operated at low and high frequency of 10 mHz and 10 kHz, respectively. At the end of the wet–dry test, the impedance of selected cycles (2, 4, 18, and 26 cycles) with an amplitude of ±10 mV was measured under frequency range of 1 mHz–100 kHz. The R_s_ and R_c_ were calculated by regression analysis based on the EIS model [21]. All AC impedance measurements were done with a two-electrode system, the same components and same exposed surface to electrolyte. 

## 3. Results

### 3.1. Corrosion Behavior of Wet–Dry Cycle

**Figure 3:**
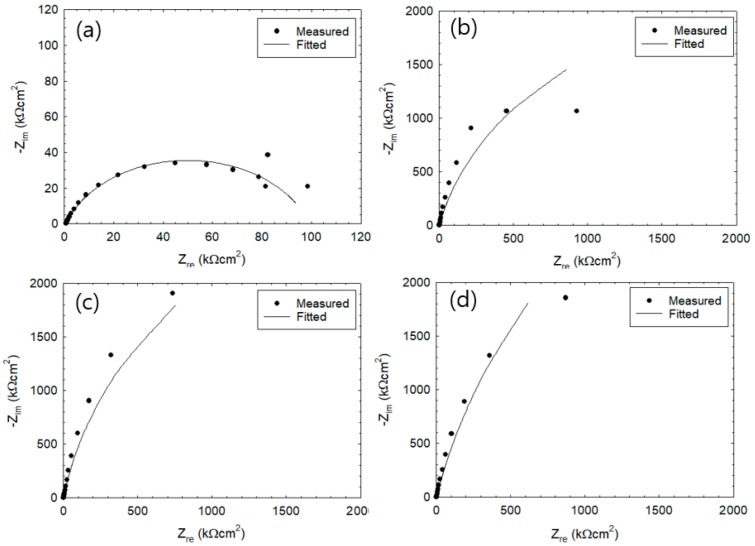
Cole–Cole plot of reinforced steel during the 2nd wet–dry cycle with cover thickness of (**a**) 5 mm, (**b**) 10 mm, (**c**) 30 mm, and (**d**) 50 mm.

**Figure 4:**
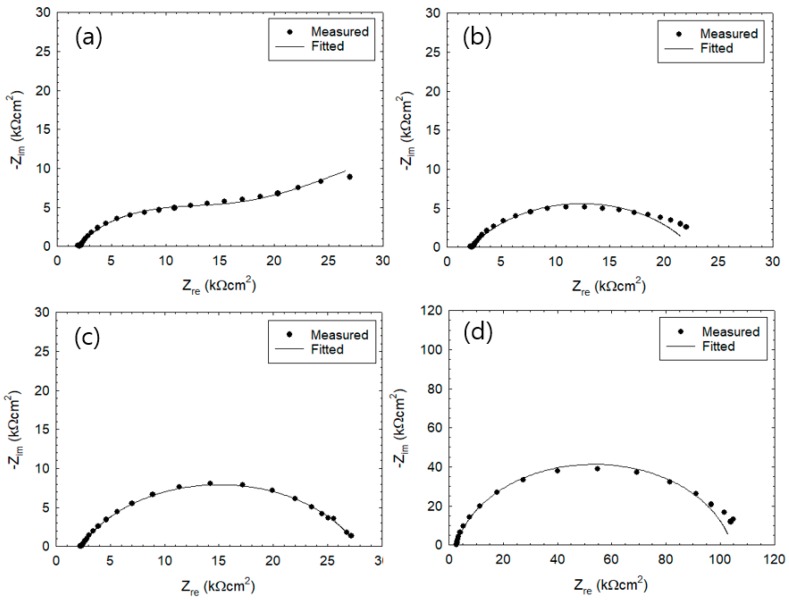
Cole–Cole plot of reinforced steel during the 26th wet–dry cycle with cover thickness of (**a**) 5 mm, (**b**) 10 mm, (**c**) 30 mm, and (**d**) 50 mm.

In Figure 3 and Figure 4, the Nyquist plots (or Cole–Cole plots) show the impedance measured after 2nd and 26th wet–dry cycle. The lines represent the fitting results. In the equivalent circuit depicted in Figure 1, R_c_ represents the charge transfer resistance, R_s_ the solution resistance, CPE the constant phase element, and W the Warburg impedance of diffusion. Table 2 summarizes values of parameters used for the fitting. CPE parameters are represented by T and α_1_. When α_1_ = 1, CPE would become capacitive, being equal to the electric double layer capacitance C_dl_ (µF/cm^2^). Impedance Z_CPE_ of CPE is represented by

(5) $$Z_{\mathrm{CPE}}=\frac{1}{\left[T{\left(j\omega \right)}^{\alpha 1}\right]},$$

where j refer to the complex and ω is the angular frequency. That is, impedance, consisting of a parallel connection of R_c_ and CPE, would yield a semi-circle trace when α_1_ = 1 and, as the α_1_ value becomes smaller, the semi-circle would become increasingly distorted. Warburg impedance W is composed of a resistance component W_R_, a capacitance component W_T_, and a calibration factor α_2,_ Provided that the mode of diffusion was finite, impedance Z_W_ of W is represented by

(6) $$Z_{W}=\frac{\mathrm{WRtanh}\left[{\left(\mathrm{jWT}\omega \right)}^{\alpha 2}\right]}{{\left(\mathrm{jWT}\omega \right)}^{\alpha 2}},$$

when α_2_ = 0.5, impedance of diffusion yields a straight line with 45° over the Nyquist plot and, with further decrease of α_2_, the slope of the straight line becomes smaller. For now, exact physical significances of the parameters, α_1_ and α_2_, are not clear but these parameters certainly refer to some aspects of electrode reaction heterogeneity. The optimum EIS curve derived from EIS equivalent circuit (Figure 1) is represented by a line and was fitted with the experimental Nyquist plot [7]. The experimental data were fitted with the theoretical curves generated by Equations (5) and (6). Results show that experimental data during 26th cycle are in good agreement with the fitted curves. Meanwhile, experimental results from 2nd cycle do not agree well with the fitted curves. The Nyquist plot at Figure 3a showed one large semi-circle while Figure 3b–d showed similar behavior where the initial state showed half of a large semicircle. After the 25th cycle of wet–dry exposure test (Figure 4a–d), the overall size of the semicircle has significantly reduced in size. Figure 4a shows a semi-circle with gentle slope that increases toward the right. The values of Z_re_ were observed to decrease as the monitoring progressed from 2nd cycle (Figure 3) to 26th cycle (Figure 4).

The present work used test specimens that have been cured for 21 days and demolded. The R_s_ values during 1st cycle showed higher value within the range of 1–2 kΩcm^2^, which is due to Ca(OH)_2_ produced by mortar hydration. However, the penetration of chloride ions into the mortar would result in rapid decreases in the resistance of the solution.

Table 2 shows the electrochemical impedance parameters such as R_s_, C_dl_, R_c_, and W that were obtained by fitting the model. The values of R_c_ were found within the range from 119 to 15,000 kΩcm^2^ at 2nd cycle and were observed to decrease significantly at the 26th cycle (R_c_ = 16–101 kΩcm^2^). The specimen with 5 mm thickness during the 26th cycle was observed to have the R_c_ values reduced to 16.9 kΩcm^2^ where the Nyquist plot showed a distorted semicircle shape within the low frequency range. The R_s_ values were determined to be within the range of 0.7–1.0 kΩcm^2^ and 2.0–2.6 kΩcm^2^ after the 1st and 26th cycle, respectively. 

The α_1_ values of the CPE in the 2nd cycle under immersion state were determined to be in the range of 0.79–0.91, which is close to 1. On the other hand, the T value in the initial state can be considered as C_dl_. With increasing cycle, the T value was observed to increase while α_1_ value was observed to decrease. The trend became more pronounced as the mortar thickness becomes thinner. Figure 3 and Figure 4b–d show the rebar surface at passive stage and low corrosion rate, which is controlled by the charge transfer step. In Figure 4a, the corrosion rate is controlled by the mass transfer step with Warbug diffusion impedance.

### 3.2. Measurement of R_p_ Via the Two-Frequency Method

The two-frequency impedance method is applied to quickly measure the corrosion rate of rebar embedded in concrete exposed to wet–dry cycles. In general, measurement of the corrosion rate of metal via AC impedance method would utilize a sample size of 5 point/decade within a high frequency region that corresponds to the solution resistance (R_s_) and low frequency region that corresponds to the charge transfer resistance (R_c_). The measurement at low frequency region takes a longer time to complete, which renders it difficult to quickly measure changes in corrosion rate caused by varying wettability of the reinforced concrete. 

**Figure 5:**
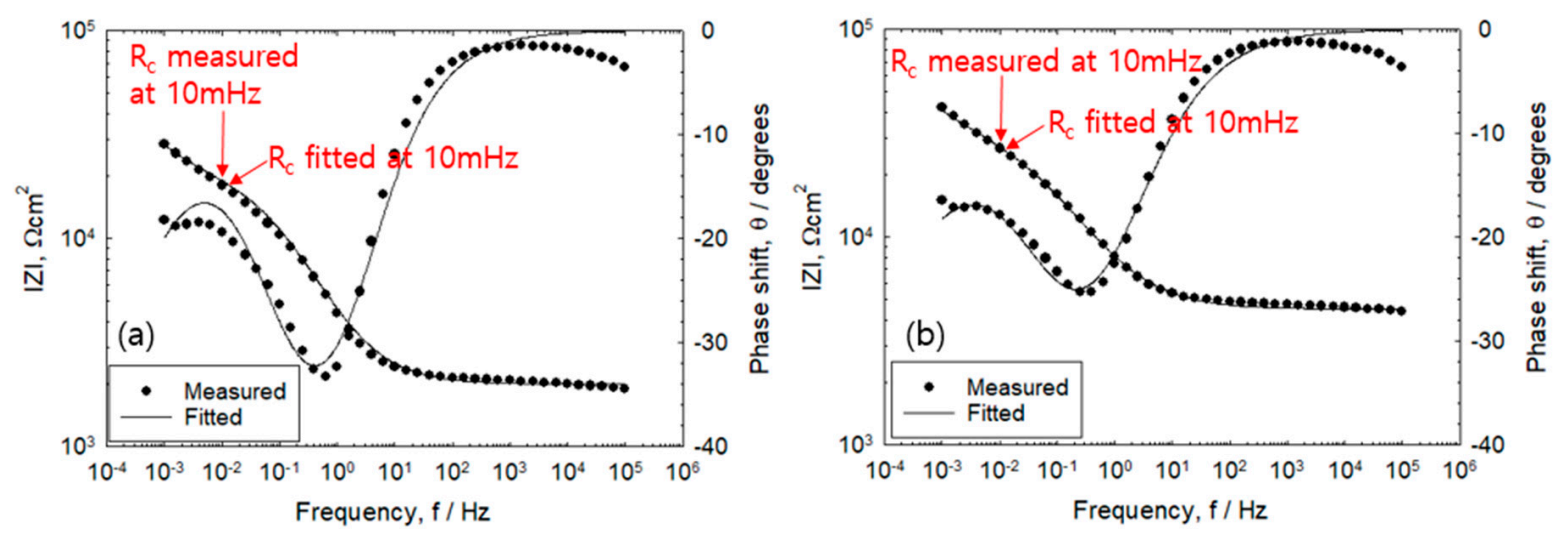
Bode diagram of reinforced concrete in the 18th cyclic with cover thickness of 5 mm measured under (**a**) immersed and (**b**) dry condition.

A comparative study was conducted in the wet–dry environment for the selection of the appropriate frequency for R_c_ and R_s_. As shown in Figure 5, bode plots illustrate the absolute value of the impedance measured during the 18th wet–dry cycle. Under wet condition, measurement of impedance started after 2 h of immersion while measurement of the AC impedance under dry conditions started after 12 h. In the low frequency region, high R_c_ values were obtained under dry condition when compared to the immersed condition. The difference in maximum phase shift is between 1 and 0.1 Hz, which is equivalent to the EIS circuit. The generated current flows on R_s_ + R_c_ at frequency range of 0.1–1.0 Hz with voltage of ±10 mV. Therefore, the frequency lower than 0.1–1.0 Hz was selected that would correspond to R_c_. Table 3 compares the results of the AC impedance measurement using 5 mm mortar thicknesses against EIS fittings. It can be seen that as the number of wet–dry cycles increases, there is also a corresponding increase in the corrosion rate while the error (%) was observed to decrease [14].

### 3.3. Monitoring of Corrosion Rate

**Figure 6:**
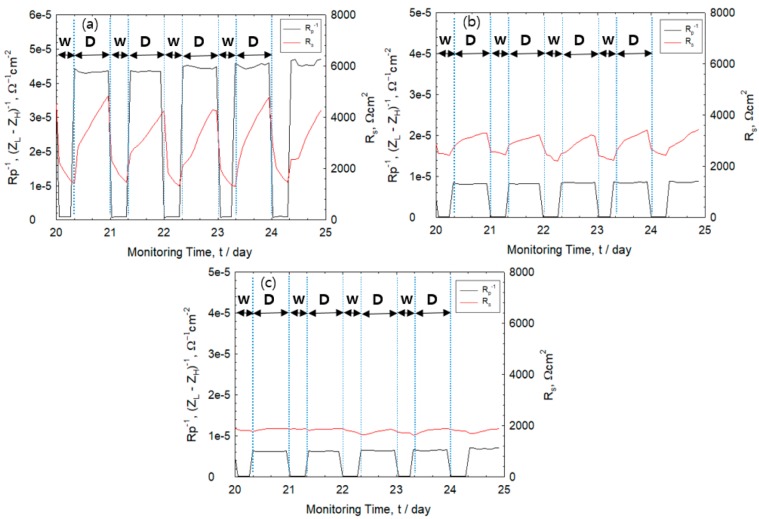
Monitoring of polarization resistance (R_p_^−1^) and solution resistance (R_s_) measured from 20th to 25th wet (**W**)–dry (**D**) cycle with cover thickness of (**a**) 5 mm, (**b**) 10 mm, and (**c**) 30 mm.

Figure 6 shows the change in the values of R_s_ and R_p_^−1^ during the period of 20th–25th cycle. The solution resistance of the rebar-mortar interface was measured using 10 kHz in the high frequency region. Meanwhile, the corrosion rate (R_p_) was computed from R_p_ = R_c_ − R_s_. The value of R_c_ is measured in the low frequency region at 10 mHz. The values of R_p_^−1^ for all specimens were observed to increase rapidly as it changes from the wet to the dry state and the values decreased rapidly from dry to wet state. A cover thickness of 30 mm resulted in relatively constant values of R_s_ for both the wet and dry state. On the other hand, an increase in R_s_ values was observed as drying progressed at cover thickness of 5 and 10 mm. Results show the importance of measuring the electrochemical parameters in the dry state in order to accurately measure the corrosion rate of reinforced concrete structures exposed to repeated wet–dry saline environments.

**Figure 7:**
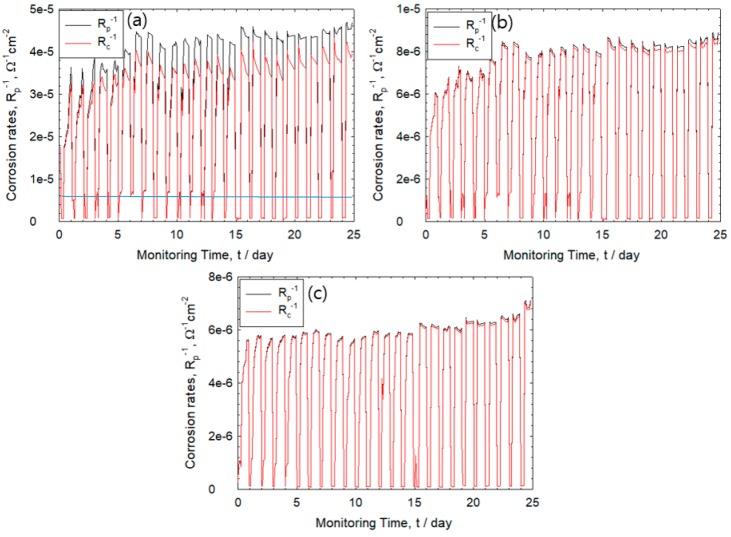
Corrosion monitoring of R_p_^−1^ (black line) and R_c_^−1^ (red line) in the 1st–25th wet–dry cycle with cover thickness of (**a**) 5 mm, (**b**) 10 mm, and (**c**) 30 mm.

Figure 7 shows the values of corrosion rates and R_s_ obtained for the 1st–25th cycle for cover thickness of 5, 10, and 30 mm. The R_p_^−1^ is expressed as the reciprocal of the polarization resistance and defined as an index of corrosion rate [16]. It was observed that there was an increase in the corrosion rate for all specimen wherein the highest rate of corrosion was displayed by rebar with 5 mm mortar thickness. The corrosion rate can be classified into four types: passivation region (R_p_^−1^ ≤ 4 × 10^−6^ Ω^−1^cm^−2^), low/middle (4 × 10^−6^ Ω^−1^cm^−2^ < R_p_^−1^ ≤ 4 × 10^−5^ Ω^−1^cm^−2^), high (4 × 10^−5^ Ω^−1^cm^−2^ < R_p_^−1^ ≤ 4 × 10^−4^ Ω^−1^cm^−2^), and very high (4 × 10^−4^ Ω^−1^cm^−2^ < R_p_^−1^ ≤ 4 × 10^−3^ Ω^−1^cm^−2^) [22,23]. The corrosion rate of rebar with cover thickness of 5 mm is classified as mid-range type while other specimens are categorized to have low corrosion rates. In Figure 7a–c, the red solid line does not take into account the value of R_s_ from R_c_ while the black solid line shows R_p_ attained by R_s_ corrected from R_c_. At a cover thickness of 5 mm, high corrosion rate implies greater rust formation that would result in high error values.

As seen in Figure 7a, thickness of 5 mm is characterized by relatively high corrosion rates and increasing values of R_s_ in the dry state. This indicates that the corrosion rate may be miscalculated if R_s_ is not corrected. Meanwhile, a relatively large mortar thickness of 10 and 30 mm displayed low corrosion rates (Figure 7b,c). In general, DC-based polarization resistance method is widely applied in the accurate measurement of corrosion rate. The method mainly determines the R_c_ value and has difficulty in measuring R_s_ values. However, higher corrosion rates and changes in R_s_ such as exposure to its drying state and formation of rust products at the rebar-mortar interface could lead to imprecise measurements [24,25]. In contrast, the two frequency-based AC method can easily correct the effect of R_s_ value and accurately measure the corrosion rate.

### 3.4. Effect of IR Drop on Corrosion Rate

In general, the corrosion rate of reinforced steel in concrete that is repeatedly exposed to wet–dry cycles such as splash zones is greater than consistent exposure to either wet or dry environment. The electrochemical changes in reinforced concrete under repeated wet–dry cycle was evaluated and discussion on the limitations in the field measurement via the EIS method was carried out.

**Figure 8:**
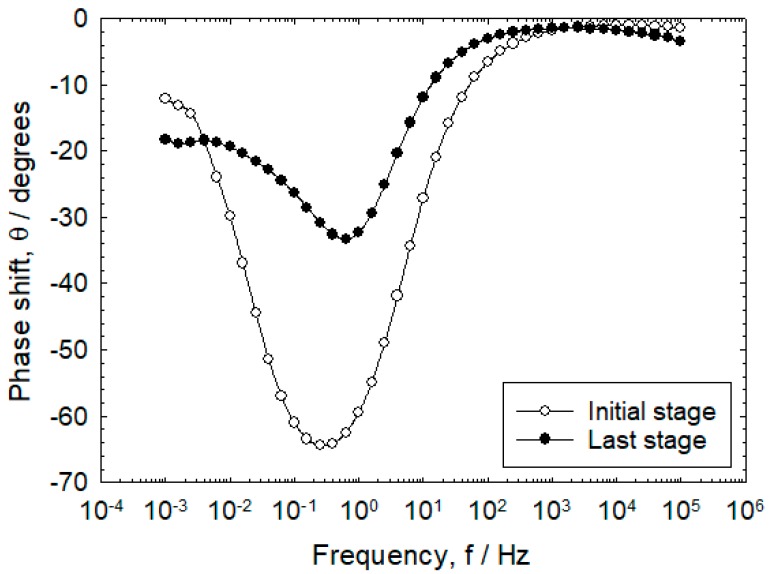
Variation of phase shift with frequency on cover thickness of 5 mm in the immersion condition at the 4th and 18th cycle.

In Figure 8, the bode plot illustrates the impedance phase difference between incoming voltage and output voltage in the 4th and 18th cycle. The EIS impedance was measured continuously in the frequency range of 100 kHz–1 mHz. Results show that 10 kHz is applicable for R_s_ value. The frequency selected that corresponds to R_s_ + R_c_ was at a frequency lower than 1–0.1 Hz [14]. When the maximum phase shift that represents the electric double layer exceeds θ = −45° under any frequency, it is known that the current line distribution measured in the frequency region lower than the arbitrary frequency is uniform [14].

Table 4 illustrates the R_s_, T, and R_c_ values of 4th and 18th cycle. The progress from 4th–18th cycle shows an increase in the values of R_s_ and T and decrease in R_c_ values. This is attributed to the penetration of chloride into the mortar that destroys the passivation film on the rebar surface and accelerates rust formation.

### 3.5. Effect of Corrosion Product on IR Drop

Figure 8 shows the results of the AC impedance measurements on the initial (4th) and last (18th) stage of the wet–dry state of rebars with 5 mm mortar thickness. It was observed that less corrosion products have formed on the rebar surface at the 4th cycle when compared to 18th cycle. Particularly, the rebar surface in the 18th cycle has a high corrosion rate that would result in greater volume of corrosion products formed. The maximum phase shifts were observed to occur from −65 to −35°, which is attributed to the non-uniformity of errors on the IR drop caused by corrosion products.

### 3.6. Effect of Drying Condition on IR Drop

As shown in Figure 8, comparing the maximum phase shift of the initial and last stage, the phase shift was observed to rapidly decrease to −35° at 18th cycle. At mortar thickness of 5 mm, high quantity of corrosion products were generated and the maximum phase shift was observed to decrease to θ = −25° during the drying process.

**Figure 9:**
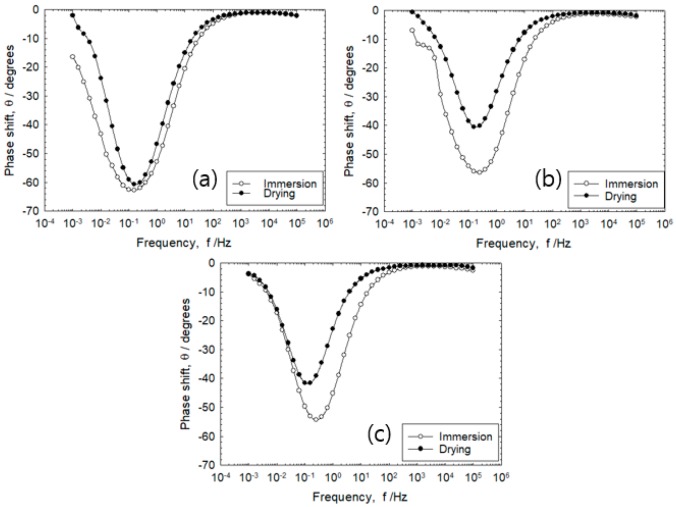
Effect of phase shift on the frequency during the 5th wet–dry cycle under a distance of (**a**) 10 mm, (**b**) 30 mm, and (**c**) 80 mm between reinforcing steels as two electrodes.

Figure 9 shows the impedance characteristics measured after 5th cycle of immersion and drying. Regardless of the distance between the two electrodes, the current line distribution remains relatively constant because the maximum phase shift is around θ = −60° during immersion. When the specimen of 30 mm and 80 mm is in a dry state, the maximum phase shift was observed to rise rapidly to θ = −45° or less. In the dry state, the R_s_ value was observed to increase from 2.3 to 5.8 kΩcm^2^ as the rebar distance was increased from 10 to 80 mm, respectively. The difference in R_s_ value was 3.5 kΩcm^2^. On the other hand, the immersion state achieved R_s_ values of 1.1 kΩcm^2^ for 10 mm and 1.7 kΩcm^2^ for 80 mm with a difference of 0.6 kΩcm^2^, which was about 5.7 times lower than in the dry state. This was in agreement with the results that R_s_ sharply increased during the drying process when compared during immersion, which caused the IR drop. Hence, it has been considered to cause inaccuracy in the monitoring of the corrosion rate via the linear polarization resistance measurement method by direct currents [15].

## 4. Discussion

The good fit of Nyquist plots at 26th cycle implies that Equations (5) and (6) can adequately represent the results due to the presence of thinner electrolyte layer [13]. In the dry state, high R_p_^−1^ values and high R_s_ values would yield lower polarization resistance and accelerated mass transport rate of dissolved oxygen at the rebar-mortar interface, which would result in higher corrosion rates [10,14,16,17,26]. This implies that the process of the rebar-mortar interface (in its completely dried up state) would cause an increase in the rate of corrosion.

The decrease in R_s_ values as wet–dry cycle increases from 1st to 25th cycle could be attributed to the dissolution of the passivation film that would result in greater salt infiltration and increase in the mass transport rate of dissolved oxygen within the mortar [27,28]. Meanwhile, a corresponding increase in the R_s_ values was observed as the number of cycles increased. This implies that the corrosion of rebar proceeded as the wet–dry cycles were repeated, which resulted in an increase in electrical resistance due to precipitation of corrosion products within the pores that exists at the rebar–mortar interface [16]. Although this value is not directly related to the corrosion of rebars, the value can be used as an environment indicator for steel corrosion. The T value increases while α_1_ value decreases as the wet–dry cycle increases due to the non-uniformity of the rebar surface caused by the adhesion of corrosion products [10]. During the dry cycle, high corrosion rates (R_p_^−1^) were observed due to the formation of oxide layers. Meanwhile, R_s_ values increased as a result of the removal of bulk solution at the onset of the drying cycle. The increasing R_s_ value is also attributed to the presence of thinner electrolyte layers during drying [28].

Based on the transmission line circuit behavior proposed by Nishikata et al. (1996), the changes in phase shift from −65° at the 4th cycle to −35° at the 18th cycle implies that there is an increase in rust formation. This would mean corrosion rates are higher at 18th cycle in comparison to the 4th cycle [13].

The constant values of corrosion rate and R_s_ attained at cover thickness of 30 mm implies that a thicker cover of the mortar requires a longer drying time for the concrete surrounding the rebars. Meanwhile, high corrosion rates were observed for cover thickness of 5 mm followed by 10 mm and 30 mm. Decreasing mortar thickness would imply higher rates of moisture evaporation and salt penetration that would result in higher corrosion rates. The higher corrosion rate in the drying period is caused by the increased reduction rate of dissolved oxygen [26]. In the dry stage, higher rebar distance of 30 and 80 mm causes a rapid rise in phase shift. This signifies that increasing the distance between electrodes would result in uneven current distribution due to drying of the mortar pores between the rebars [17].

## 5. Conclusions

The present study evaluated the monitoring of reinforced concrete via electrochemical method. The specimens were repeatedly submerged in NaCl solution and air-dried for a total of 25 wet–dry cycles, and the following conclusions were made:

1. During the early stage of wet–dry cycle, the rebar/mortar is represented by a simple equivalent circuit. As the wet–dry cycle progresses, chloride ion penetration would cause the charge transfer resistance to decrease. Meanwhile, the Warbug impedance that represents oxygen diffusion in the mortar was detected in the low frequency range.
2. During the drying stage, both corrosion rate and R_s_ of the rebars were observed to increase rapidly. The R_s_ value was observed to become higher under dry condition, which is attributed to thinner electrolyte layers and higher corrosion rate of the rebar. The high corrosion rate indicates formation of oxide layers during the drying cycle.
3. During monitoring of the corrosion rate via AC impedance, accurate measurement of the polarization resistance was ensured by compensating the IR drop based on the dry state of the specimen and quantity of corrosion products found on the rebar surface.
4. The value of the polarization resistance corrected using R_s_ was different from the polarization resistance without R_s_ correction, attributed from drying condition, rust layer, distance between electrodes.
5. The percent error (%) of the charge transfer resistance and polarization resistance derived from the fitting of Nyquist plots with the experimental results at 10 mHz/10 kHz was observed to decrease with increasing wet–dry cycles.

## Figures and Tables

**Table 1:** Experimental parameters applied in specimen preparation.

Specimen	Clear Cover (mm)	Distance of Rebar (mm)
1	5	10
2	10	10
3	30	10
4	50	10
5	10	10
6	10	30
7	10	80

**Table 2:** Values of corrosion constants derived from the 2nd and 26th wet–dry cycle.

Parameters	Wet Process in 2nd Cycle				Wet Process in 26th Cycle			
	Clear Cover (mm)				Clear Cover (mm)			
	5	10	30	50	5	10	30	50
R_s_ (kΩcm^2^)	0.8	0.7	1.0	0.9	2.0	2.2	2.2	2.6
T (μFcm^−2^)	63	44	48	45	98	83	84	45
R_c_ (kΩcm^2^)	119	500	14,284	15,000	16.9	20.3	25.5	101.5
α_1_	0.79	0.88	0.91	0.89	0.64	0.64	0.71	0.87
W_R_ (Ωcm^2^)	-	-	-	-	440	-	-	-
W_T_	-	-	-	-	752	-	-	-
α_2_	-	-	-	-	0.18	-	-	-

**Table 3:** Comparison of experimental values with the theoretical values derived from EIS fittings of charge transfer resistance and solution resistance measured at 10 mHz/10 kHz.

Cycles		Experimental			Fitted			Error (%)		
		R_c_	R_s_	R_p_	R_c_	R_s_	R_p_	R_c_	R_s_	R_p_
**2**	Wet	79.7	0.8	78.9	119.8	0.8	119.0	33.4	0	33.6
4	Wet	66.4	0.6	65.7	99.9	0.6	99.2	33.0	0	33.0
	Dry	57.5	2.4	55.0	66.0	2.4	63.6	12.9	0	13.4
18	Wet	18.1	1.9	16.1	17.5	1.9	15.5	3.4	0	3.9
	Dry	26.7	4.5	22.1	25.7	4.5	21.1	3.8	0	4.7
26	Wet	17.9	1.9	15.9	16.9	2.0	14.8	6.0	0	7.0

**Table 4:** Values of corrosion constants derived from the 4th and 18th wet–dry cycle.

Parameters	Wet Process in 4th Cycle		Wet Process in 18th Cycle	
	Clear Cover (5 mm)		Clear Cover (5 mm)	
	Experiment	Fitted	Experiment	Fitted
R_s_ (kΩcm^2^)	0.6	0.6	1.9	1.9
T (μF(cm^−2^))	-	71	-	88
R_c_ (kΩcm^2^)	66.4	99.9	18.1	17.5
α_1_	-	0.77	-	0.69
W_R_ (Ωcm^2^)	-	-	-	370
W_T_	-	-	-	600
α_2_	-	-	-	0.23
